# Comparison of Postoperative Opioid Consumption of Paravertebral Block and Erector Spinae Plane Block After Thoracotomy: A Randomized Controlled Trial

**DOI:** 10.7759/cureus.59459

**Published:** 2024-05-01

**Authors:** Mustafa Duran, Alparslan Kuş, Can Aksu, Sevim Cesur, Hadi Ufuk Yörükoğlu, Tulay Hosten

**Affiliations:** 1 Anesthesiology and Reanimation, Marmara Üniversitesi Pendik Eğitim Araştırma, Istanbul, TUR; 2 Anesthesiology and Reanimation, Kocaeli University, Kocaeli, TUR; 3 Anesthesiology, Kocaeli University, Kocaeli, TUR; 4 Anesthesiology, Kocaeli University School of Medicine, Kocaeli, TUR; 5 Anesthesiology and Reanimation, Kınık State Hospital, Izmir, TUR; 6 Anesthesiology and Reanimation, Faculty of Medicine, Kocaeli University, Kocaeli, TUR

**Keywords:** thoracic anesthesiology, regional anesthesiology, erector spinae plane block (espb), thoracic paraverterbral block, postoperative morphine use

## Abstract

Background

Thoracotomy is associated with severe postoperative pain. Pain developing after thoracotomy causes lung infections, inability to expel secretions, and atelectasis as a result of deep breathing. Effective management of acute pain after thoracotomy may prevent these complications. A multimodal approach to analgesia is widely employed by thoracic anesthetists using a combination of regional anesthetic blockade and systemic analgesia, with both non‐opioid and opioid medications and local anesthesia blockade. Nowadays, regional anesthesia techniques such as thoracic epidural paravertebral block (PVB), erector spinae plane block (ESPB), and serratus plane block are frequently used to prevent pain after thoracotomy. In this study, we compared paravertebral block with erector spinae block for pain relief after thoracotomy. Our primary aim was to determine whether there was a difference between postoperative opioid consumption and pain scores. We also compared the two regional anesthesia techniques in terms of intraoperative hemodynamic data and postoperative complications.

Methodology

Patients aged between 18 and 75 years with an American Society of Anesthesiology (ASA) physical status I-III and scheduled for elective thoracotomy were included in the study. Using www.randomizer.org, patients were divided into two different groups, namely, ESPB and PVB. All patients were provided with a patient-controlled analgesia device preloaded with morphine. Postoperative 24-hour morphine consumptions were recorded.

Results

Data from 45 patients were used in the final analyses. Morphine consumption was higher in the ESPB group than in the PVB group at 24 hours postoperatively (19.2 ± 4.26 mg and 16.2 ± 2.64 mg, respectively; p < 0.05). There was no significant difference in numerical rating scale scores both at rest and with coughing (p > 0.05). Intraoperative heart rates were similar between groups. However, mean intraoperative blood pressure was significantly lower in the PVB group at 30 minutes (p < 0.05). Nausea and vomiting were observed in two patients in the ESPB group and one patient in the PVB group. The complication of nausea and vomiting was not statistically significant between the two groups (p > 0.05). Catastrophic complications such as hematoma, pneumothorax, and local anesthetic systemic toxicity were not observed in either group.

Conclusions

We found that patients who underwent PVB consumed less morphine postoperatively than patients who underwent ESPB. However, we did not observe any difference in pain scores between both groups. We think that ESPB can be considered a reliable method in thoracotomy surgery due to its ease of application and the fact that the place where the block is technically performed is farther from the central structures compared to PVB. In light of the results of our study, ESPB can be used as an alternative to PVB, which has been proven as postoperative analgesia in thoracic surgery.

## Introduction

Postoperative pain management aims to minimize hospital stays, promote patient comfort, and prevent the negative effects of pain [1]. Thoracotomy is associated with extremely painful postoperative discomfort [2]. After thoracotomy, pain can be managed using a variety of techniques. The most effective method of pain management is considered to be thoracic epidural analgesia [3]. However, facial blocks are becoming increasingly popular due to the serious side effects of epidural analgesia.

Studies have shown that paravertebral block (PVB) after thoracotomy provides effective, unilateral, somatic, and visceral analgesia [3]. PVB has a lower risk of complications and reduces post-thoracotomy pain similar to epidural analgesia [1,4]. A new fascial plane block technique, i.e., erector spinae plane block (ESPB) [5], has been shown to be effective in reducing pain during thoracotomy [6]. ESPB has a lower risk of complication and is safer and easier to perform [7]. ESPB reduces the risk of bleeding and hematoma because it is applied more superficially than PVB and epidural analgesia and is further from major arterial systems and the pleura. As a result, it can be used safely in combination with anticoagulants [8].

The main aim of our study was to compare PVB, ESPB, and postoperative opioid use in thoracotomy patients. Comparing the ease of administration, intraoperative hemodynamic effects, rest and cough, numerical pain scores (NRS), and complications of PVB and ESPB was our secondary objective.

## Materials and methods

**Figure 1:**
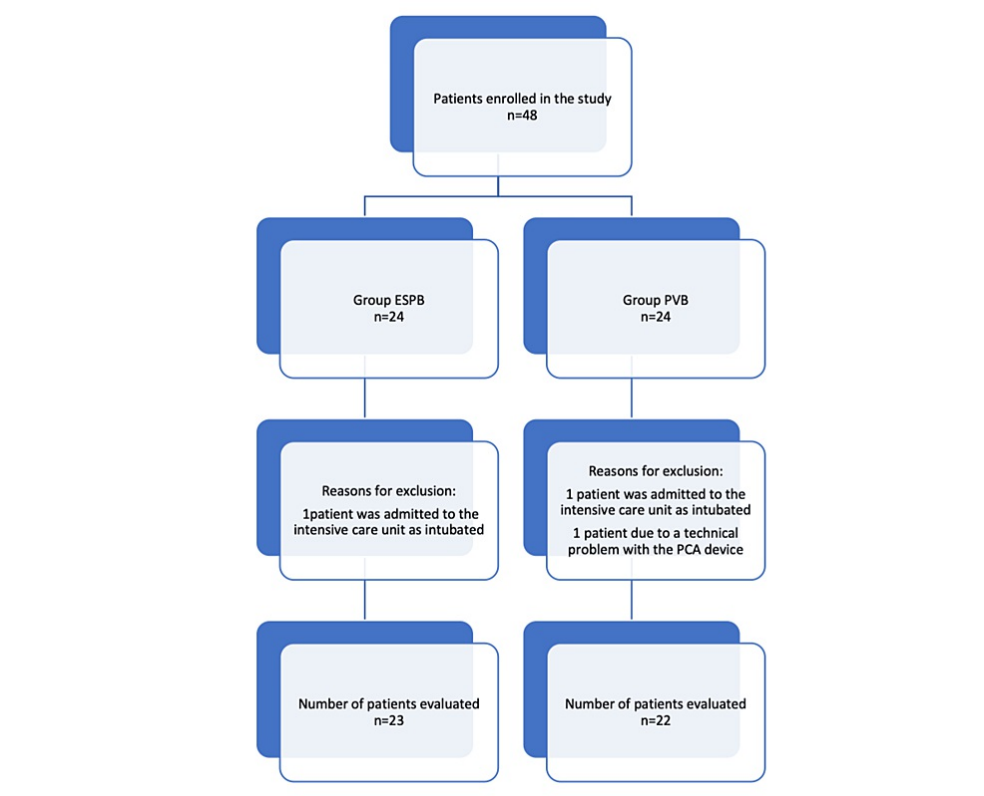
CONSORT flow diagram.

This trial was conducted at Kocaeli University Medical Faculty Hospital among patients undergoing elective posterolateral thoracotomies. The ethics committee of Kocaeli University Medical Faculty approved the trial (approval number: GOKAEK-2020/287), which was conducted in accordance with the Declaration of Helsinki and the Guideline for Good Clinical Practice. The trial was registered at Clinicaltrials.gov (registration number: NCT04999319). Written informed consent was obtained from each participant before participation in this trial. The Consolidated Standards of Reporting Trials (CONSORT) flowchart was used to allocate patients to the trial (Figure 1).

The study enrolled patients aged 18 to 75 years who were scheduled for elective unilateral thoracotomy and had American Society of Anesthesia (ASA) class I-III physical status. The study included patients undergoing posterolateral thoracotomy for lung resection. Exclusion criteria included obesity (body mass index >35 kg/m^2^), skin infection at the site of the needle puncture, known drug allergy, coagulopathy, recent opioid use, inability to understand or use the numeric rating pain scoring system, or a patient-controlled analgesia (PCA) pump.

Using the computerized randomization method (https://www.randomizer.org), patients who agreed to participate in the trial were randomized to one of two groups. The trial was conducted in a blinded fashion by a registered pain nurse who was unaware of which block was given to which patient.

Approximately one hour before the procedure, all patients were taken to the block room and fitted with standard non-invasive monitoring devices, including electrocardiography (ECG), peripheral oxygen saturation, and non-invasive blood pressure. Patients were premedicated with 50 mg of fentanyl and 0.03 mg of midazolam per kilogram. Patients in the PVB group underwent PVB and patients in the ESPB group underwent ESPB. An experienced practitioner (with more than 50 applications for each block) performed all blocks.

All blocks were performed using the Esaote My Lab 6 US machine (Florence, Italy) equipped with a 22 G, 50 mm, Braun Sonoplex (Melsungen, Germany) nerve block needle and a wide bandwidth, multi-frequency linear probe (2.5-12.5 MHz). The T4 vertebra was identified by counting from the C7 vertebra by physical examination, while the corresponding dermatome area was determined.

ESPB technique

With the patient in the prone position, a unilateral ESPB block was applied at the level of the T4 vertebra. The linear probe was positioned 2 cm lateral to the spine using a sagittal approach and the erector spinae muscle was located above the transverse processes of the spine. The needle was inserted craniocaudally, penetrating deep into the erector spinae muscle. Correct placement of the needle tip was achieved by administering 0.5-1 mL of normal saline. Then, 20 mL of 0.5% bupivacaine was administered for the block. The local anesthesia distribution was visible in both cranial and caudal directions.

PVB technique

After proper skin cleansing, the block was administered with the patient in the prone position. At the T4 level, a linear ultrasound probe (Esaote My Lab 6, Florence, Italy) was positioned parallel to the spine and moved 2 cm laterally. The paravertebral space, transverse process, and pleura were visible. A caudal to cranial direction was used to place the needle using an in-plane technique. At the T4 level, 20 mL of 0.5% bupivacaine was injected into the paravertebral space.

The effectiveness of each block was assessed every five minutes by a pinprick test with loss of chest wall sensation. By comparison with the contralateral side, the blocked dermatome area was identified first in the cranial direction and then in the caudal direction.

All patients received standardized monitoring in the operating theater, including non-invasive blood pressure, SpO_2_, and ECG monitoring. Propofol (2-3 mg/kg), fentanyl (1 g/kg), and rocuronium (0.6 mg/kg) were administered intravenously to induce general anesthesia and relax muscles before intubation. A 2:1 mixture of sevoflurane, oxygen, and fresh gas flow was used to maintain anesthesia, together with a remifentanil infusion. Bi-spectral index and exhaled sevoflurane concentration were used to measure the depth of anesthesia during titration of the remifentanil infusion.

Patients received 1 g of paracetamol, 0.05 mg/kg morphine, and 20 mg of tenoxicam approximately 30 minutes before the end of surgery. In addition, 8 mg of ondansetron was administered to prevent postoperative nausea and vomiting. The postoperative intensive care unit received the extubated patients during the recovery period and was responsible for their care. A PCA device with morphine met the analgesic requirements of all patients. Patient-controlled analgesia with morphine was set at a concentration of 0.5 mg/mL in a volume of 100 mL, a 2 mg bolus, an eight-minute lock-in period, and 6 pressures per hour. A pain nurse, blinded to which procedure was being performed for which patient, assessed the patient’s pain and graded it using the NRS. A pain technician, unaware of which group the patients belonged to, recorded the patient’s resting and coughing NRS scores (no pain: 0, excruciating pain: 10), morphine consumption, blood pressure, heart rate and saturation levels, nausea, and any complaints such as vomiting. Morphine was planned to be administered if the NRS score of 4 and above did not meet the hourly morphine consumption limits (6 mg of morphine), and if the hourly morphine consumption limits were exceeded, 1 g of paracetamol was administered as rescue analgesia. If the NRS score of 4 or above did not meet the hourly morphine limit (6 mg), morphine was administered. If the hourly morphine limit was exceeded, 1 g of paracetamol was given as rescue analgesia.

Statistical analysis

The sample size for our study was calculated according to the study by Biçer et al. [9]. Considering our primary aim, opioid consumption in the first 24 hours postoperatively, the sample size for each group was calculated as 22 patients, with a margin of error of 0.05 and a power of 0.80, and 24 patients were included in each group. Statistical evaluation was done using SPSS version 20.0 (SPSS Inc., Chicago, IL, USA). The normal distribution test was evaluated with the Kolmogorov-Smirnov test. Numerical variables were presented as mean ± SD, median (25th-75th percentile), and frequency (percentiles). The difference between the groups was evaluated using Student’s t-test for numerical variables with normal distribution and the Mann-Whitney U test for those without normal distribution. Relationships between categorical variables were evaluated using the chi-square test. P-values <0.05 were considered statistically significant.

## Results

A total of 48 patients were enrolled in this study. The CONSORT diagram of the trial enrolment is shown in Figure 1. The final analyses included data from 45 patients. The ASA classifications and demographic characteristics of the patients in both groups were comparable. Patients included in the study were matched for surgical procedures, anesthetic and operative time, and intraoperative remifentanil consumption. There were no significant differences between the groups (Table 1).

**Table 1:** Demographic data. Data are expressed as mean ± standard deviation or number. ESPB = erector spinae plane block; PVB = paravertebral block; BMI = body mass index; ASA = American Society of Anesthesiology

	Group ESPB (n = 23)	Group PVB (n = 22)	P-value
Age (years)	56.1 ± 13.0	55.4 ± 14.1	0.860
Sex			0.486
Female	7	4	
Male	16	18	
BMI (kg/m^2^)	25.9 ± 4.3	26.1 ± 4.0	0.830
ASA			0.195
I	6	6	
II	10	5	
III	7	11	
Type of surgery			0.698
Lobectomy	21	21	
Segmentectomy	1	0	
Pneumonectomy	1	1	
Duration of surgery (minute)	219 ± 21.2	220.8 ± 23.6	0.790
Intraoperative remifentanil consumption (µg)	940.9 ± 450.8	803 ± 638.4	0.413

Postoperative morphine consumption was 19 mg in the ESPB group and 16 mg in the PVB group, which was statistically significant (p < 0.05) (Table 2). There was no significant difference in NRS values both at rest and with coughing (p > 0.05) (Table 2).

**Table 2:** Comparison of NRS scores and morphine consumption between groups. Data are expressed as median (percentiles 25-75). ESPB = erector spinae plane block; PVB = paravertebral block; NRS = numerical pain scores

	Group ESPB (n = 23)	Group PVB (n= 22)	P-value
NRS resting, 1st hour	4 (1.75–4)	3 (2.5–3.25)	0.898
NRS resting, 6th hour	2 (2–3)	3 (2–3)	0.179
NRS resting, 12th hour	2 (2–3)	2 (1.75–2.25)	0.164
NRS resting, 24th hour	2 (2–2.5)	2 (1–3)	0.478
Morphine consumption, 1st hour (mg)	2.5 (2–4)	2 (1–3)	0.164
Morphine consumption, 6th hour (mg)	3 (2–5)	3 (2–4.25)	0.710
Morphine consumption, 12th hour (mg)	5.5 (4–7)	4 (2.75–5.25)	0.106
Morphine consumption, 24th hour (mg)	19 (16–22)	16 (15–17.25)	0.012

Intraoperative heart rates were similar between groups. However, the mean intraoperative blood pressure was significantly lower in the PVB group at 30 minutes (p < 0.05).

Nausea and vomiting were observed in two patients in the ESPB group and one patient in the PVB group. The complication of nausea and vomiting was not statistically significant between the two groups (p > 0.05). Catastrophic complications such as hematoma, pneumothorax, and local anesthetic systemic toxicity (LAST) were not observed in either group.

## Discussion

In our study, we found that ESPB was more effective in relieving acute distress after PVB thoracotomy. In a study conducted by Fang et al. [6] in 94 patients, PVB and ESPB were shown to provide effective analgesia after thoracotomy. In this study, postoperative intravenous sufentanil PCA was administered, and resting and cough visual analog scale (VAS) scores were recorded. A comparison of VAS scores and opioid consumption of both ESPB and PVB patients showed that both blocks provided effective analgesia for thoracotomy.

In our study, the PVB group had lower opioid consumption (p = 0.05) as PCA consumption and VAS were close to each other in both groups in the postoperative period. In our study, 20 mL of 0.5% local anesthesia was used for both blocks. Zabaleta et al. [10] compared the efficacy of PVB using two different concentrations in patients undergoing thoracotomy. Although one group received 0.5% 20 mL bupivacaine and the other 0.33% 20 mL bupivacaine, there was no significant difference in postoperative VAS scores or morphine consumption between the two groups. In general, studies have found that 0.25%-0.5% in a volume of 15-30 mL bupivacaine produces acceptable analgesia, although they disagree on the concentration and volume of local anesthetic [11]. Unlike the long-used PVB, there is insufficient research in the literature to determine the correct local anesthetic concentration for the newly developed ESPB block. According to Chin et al. [12], the drug used at high concentrations was better at preventing the *fast* pain sensation that is critical for thoracotomy than the drug given as an injection for ESPB. We believe that our study will contribute to the literature by demonstrating the efficacy of using a single dose of 20 mL 0.5% local anesthetic in both blocks.

Initially described for thoracic neuropathic pain, ESPB has rapidly gained popularity due to its ease of application and potent analgesia [5,13]. Thoracic surgical techniques are now preferred over other types of surgeries due to the ability of ESPB to provide effective postoperative analgesia. In thoracotomy surgery, Sobhy et al. [14] compared ESPB with intravenous PCA. They showed that ESPB provided more effective analgesia.

Tulgar et al. [15] evaluated the analgesic efficacy of one- or two-stage ESPB using 30 mL 0.375% bupivacaine after thoracotomy surgery. They found that postoperative tramadol consumption was statistically significantly lower in the two-stage group than in the one-stage group. The sample size of both studies was limited, highlighting the need for randomized controlled trials.

Drug distribution is critical for both blocks to provide good analgesia. In a study of 10 volunteers, 20 mL of 1% mepivacaine was used for PVB at the T6 level. According to an MRI study, a local anesthetic can reach four vertebral levels along the longitudinal axis, the intercostal region laterally, the epidural region medially via the intervertebral foramen, and the contralateral paravertebral region via the prevertebral and epidural regions [16].

In cadaveric and MRI studies, 25 mL of local anesthetic was found to spread from the T5 level to the T1-11 vertebral levels in ESPB and reached the intercostal region [17,18]. This local anesthetic distribution in our study can be used to explain the effective analgesia of both blocks administered at a single level with 20 mL of local anesthetic. A shortcoming of our study is the lack of sensory evaluation, including the relevant dermatomal regions of the patients after both blocks.

When we compared blood pressure and heart rate in our study, there was no significant difference in heart rate between the groups, but mean arterial blood pressure was significantly lower in PVB than in ESPB except after intubation and at five minutes (p = 0.05). According to Purcell-Jones et al. [19], 5 mL of contrast injected into the paravertebral space remained there in only 18% of patients, migrated to the epidural space in 70%, and diffused there in 31%. However, based on the current research, it has not been conclusively proven that the administered substance will diffuse into the epidural space and cause sympathetic blockade [20].

The distance of the ESPB from neuraxial structures reduces the hemodynamic effects of the block. In this study, we hypothesize that the reason for the lower blood pressure with PVB compared to ESPB may be related to the drug diffusion mechanism, the ability of the drug to pass from the paravertebral space to the epidural space, or the concentration of 0.5% local anesthetic we used. Due to increased sympathetic activity during intubation and skin incision and the fact that the effect of the block has not yet fully started, there may be no significant difference until after intubation and up to five minutes.

Fang et al. [6] evaluated needle advancement and drug injection time and showed that the time required for ESPB was much shorter than PVB. As the paravertebral area is deeper and adjacent to the lung, PVB is a more advanced block and care should be taken when advancing the needle. Practitioners find it more difficult to perform PVB because they fear serious complications such as hematoma pleural puncture that may occur during PVB administration. In addition, in our study, blocks were performed with the patient in the prone position. We believe that PVB, which requires a high level of skill, may provide a practical advantage in the prone position to maintain the stability of the ultrasound probe.

In our study, complications such as pneumothorax, hematoma, and LAST that may lead to mortality were not observed after both blocks. Nausea and vomiting were observed in two patients in the ESPB group and one patient in the PVB group in the first 24 hours after surgery. Although not statistically significant, we encountered more nausea and vomiting in the ESPB group with approximately 3 mg more morphine consumption than in the PVB group. The incidence of complications has decreased with the development of ultrasonography technology and its use in regional anesthesia [21]. Terkawi et al. [22] compared the complication rate of anatomical marking with ultrasonography and PVB and showed that the complication rate decreased from 26% to 2.3% in blocks performed with ultrasonography.

We believe that this risk is low because the needle is simultaneously visualized as it is advanced and the transverse process is targeted. De Cassai et al. [23], in a review of thoracic ESPB performed with both approaches and ultrasonography, found the complication rate of ESPB to be less than 0.02%. In our study, all blocks were performed using the in-plane technique and were simultaneously visualized with USG during needle advancement. In addition, negative aspiration was frequently performed and the spread of the drug was monitored by gradual injection of the drug. No serious complications, such as pleural puncture, pneumothorax, bradycardia and hypotension, hematoma, and systemic complications of local anesthetics, were observed in our study.

The reduction of pain after thoracic surgery with two facial plane blocks increased patient comfort. In our study, the absence of problems with these two blocks was considered an advantage for patients. The smallest clinically significant difference is the reduction of discomfort.

One of the secondary aims of our study was to determine whether a block could be easily placed, although it may have been necessary to define the time of needle penetration and local anesthetic distribution.

## Conclusions

Both blocks may be preferred as a component of multimodal analgesia in thoracotomy surgery for postoperative analgesia. Due to its ease of use and the fact that the location of the block is further from the central structures than PVB, we believe that ESPB can be considered a reliable technique in thoracotomy surgery.

In thoracotomy surgery, ESPB also appears to provide effective postoperative analgesia. It is considered advantageous because it can be applied more quickly by the surgeon and has less impact on intraoperative hemodynamic data. We attribute the absence of problems after both blocks to the use of ultrasonography and our needle placement method. The risk of problems is reduced by performing ESPB away from the pleura and vascular structures and using the transverse process as a barrier. ESPB can be used as an alternative to PVB, which is well-established for postoperative analgesia in thoracic surgery.
